# Retraction note: Expression of Ski in the Follicles of eCG-primed Immature Hypophysectomized Rat Ovary

**DOI:** 10.1186/s40781-017-0134-8

**Published:** 2017-05-02

**Authors:** Yeoung-Gyu Ko, Dong Hun Kim, Soo Bong Park, Sung Woo Kim, Yoon Jung Do, Hyun Kim

**Affiliations:** 1 Department of Veterinary Physiology, Graduate School of Agricultural and Life Science, The University of Tokyo, 1-1-1 Yayoi, Bunkyo-ku, Tokyo, 113-8657 Japan; 2 Animal Genetic Resources Station, National Institute of Animal Science, RDA, Namwon, 590-832 South Korea

## Retraction

This article [1] has been retracted by the corresponding author, Hyun Kim, because the data reported in the article have been previously published the Journal of Reproduction and Development [2]. We have been unable to contact the other authors regarding the retraction of their article.
